# Dexamethasone Alleviates Tumor-Associated Brain Damage and Angiogenesis

**DOI:** 10.1371/journal.pone.0093264

**Published:** 2014-04-08

**Authors:** Zheng Fan, Tina Sehm, Manfred Rauh, Michael Buchfelder, Ilker Y. Eyupoglu, Nicolai E. Savaskan

**Affiliations:** 1 Department of Neurosurgery, University Hospital of Erlangen, Friedrich Alexander University of Erlangen-Nuremberg, Erlangen, Bavaria, Germany; 2 Department of Pediatrics and Adolescent Medicine, University Hospital Erlangen, Friedrich Alexander University of Erlangen-Nuremberg, Erlangen, Bavaria, Germany; University of Florida, United States of America

## Abstract

Children and adults with the most aggressive form of brain cancer, malignant gliomas or glioblastoma, often develop cerebral edema as a life-threatening complication. This complication is routinely treated with dexamethasone (DEXA), a steroidal anti-inflammatory drug with pleiotropic action profile. Here we show that dexamethasone reduces murine and rodent glioma tumor growth in a concentration-dependent manner. Low concentrations of DEXA are already capable of inhibiting glioma cell proliferation and at higher levels induce cell death. Further, the expression of the glutamate antiporter xCT (system X_c_^−^; SLC7a11) and VEGFA is up-regulated after DEXA treatment indicating early cellular stress responses. However, in human gliomas DEXA exerts differential cytotoxic effects, with some human glioma cells (U251, T98G) resistant to DEXA, a finding corroborated by clinical data of dexamethasone non-responders. Moreover, DEXA-resistant gliomas did not show any xCT alterations, indicating that these gene expressions are associated with DEXA-induced cellular stress. Hence, siRNA-mediated xCT knockdown in glioma cells increased the susceptibility to DEXA. Interestingly, cell viability of primary human astrocytes and primary rodent neurons is not affected by DEXA. We further tested the pharmacological effects of DEXA on brain tissue and showed that DEXA reduces tumor-induced disturbances of the microenvironment such as neuronal cell death and tumor-induced angiogenesis. In conclusion, we demonstrate that DEXA inhibits glioma cell growth in a concentration and species-dependent manner. Further, DEXA executes neuroprotective effects in brains and reduces tumor-induced angiogenesis. Thus, our investigations reveal that DEXA acts pleiotropically and impacts tumor growth, tumor vasculature and tumor-associated brain damage.

## Introduction

Gliomas are one of the leading causes in brain tumor-related deaths in children and humans [1] [2]. Among primary brain tumors, the most aggressive and frequent ones are malignant gliomas, i.e. high grade gliomas including malignant gliomas WHO grade III and glioblastomas, WHO grade IV. These tumors have a very poor prognosis despite of state-of-the-art multimodal treatments, including surgical resection, irradiation and chemotherapy [3]. Patients with glioblastoma have an average survival time of about 14 months [1] [4] [5]. Malignant gliomas are hypervascularized tumors which frequently come along with vasogenic and cytotoxic brain edema as a severe and life-threatening complication [6] [7]. Tumor-induced brain edema is caused by two interdependent mechanisms: Brain tumors induce abnormal angiogenesis with impaired blood–brain barrier allowing plasma to enter the interstitial space referred to as vasogenic edema [8]. Secondly, brain tumors induce neuronal cell death and neurodegeneration by which cytotoxic brain edema can be formed inducing neurological deficits and intractable seizures [6] [9]. Notably, one major cause of morbidity and death in brain tumors is the development of uncontrolled brain edema due to cerebral herniation in more than 60% of patients suffering from glioblastoma [10] [11]. Thus, inhibition of brain edema is a vital and important strategy in fighting brain tumor-associated comorbidities. Up to now, patients with brain tumors are most commonly treated with dexamethasone [12], a synthetic glucocorticoid with potent anti-inflammatory activity. Since the introduction of dexamethasone in 1962, it has become a standard treatment in brain tumor-associated cerebral edema for more than four decades [13]. Approximately 70% of malignant brain tumor patients receive dexamethasone treatment while they undergo multimodal radio-chemotherapy and a significant decrease in deaths has been related to this treatment [14]. However, although this drug has been routinely used for decades in the management of cerebral edema, its exact mechanism of action on the tumor microenvironment is not fully uncovered. It is thought that dexamethasone blocks inflammation pathways by acting on glucocorticoid receptors, thus resulting in reduction of vessel permeability of tumor capillaries and in increased extracellular fluids clearance. Despite its usefulness, dexamethasone can produce many unintended serious side effects, including Cushing's syndrome, myopathy and opportunistic infections [15] [16]. Moreover, recent studies reported that dexamethasone can potentially interfere with current standard anticancer treatments and lower their efficacies. For instance, it has been shown that dexamethasone protects glioma cells from the chemotherapeutic agent temozolomide [17] [18], reduces the bystander effect of the thymidine kinase/ganciclovir system in suicide-gene therapy [19] and inhibits the antitumor effect of interleukin-4 [20]. Overall, these findings promoted investigations of alternative edema controlling agents. Recent data showed that the glutamate/cysteine antiporter xCT is involved in brain tumor-induced edema [6] [7]. Also, anti-edema effects of VEGF-targeted therapeutic approaches have been established in preclinical models and phase I-II studies [10] [21].

In the present study we investigated the role of dexamethasone in different established glioma cell lines and its impact on the brain-tumor microenvironment. We show that dexamethasone decreases tumor-induced neuronal damage and reduces glioma cell growth in a concentration-dependent manner. However, the growth inhibitory effect of dexamethasone on gliomas is to some extent differential depending on whether the species is rodent, murine or human. DEXA inhibits rodent and murine glioma cell growth already at low concentration and does not affect the viability of primary astrocyte growth nor primary neurons. Furthermore, DEXA induces xCT and VEGFA expression in murine and rodent gliomas as early responses of cell stress. In the peritumoral brain area, DEXA treatment normalized vessel morphology and vessel density.

## Materials and Methods

### Cell lines

The rat glioma cell lines F98 and C6, mouse glioma cell line GL261 and the human glioma U87, U251 and T98G cells are provided by LGC Standards-ATTC GmbH (Wesel, Germany). All cells were cultured in T75- cell culture flask containing DMEM medium (Biochrom, Berlin, Germany) including 10% fetal bovine serum (Biochrom, Berlin, Germany), 1% Penicillin/Streptomycin (Biochrom) and 1% Glutamax (Gibco/Invitrogen, California, USA). Cells were passaged at approximately 80% confluence by adding trypsin after one wash step with PBS. Afterwards, cells were incubated for 5 min, then centrifuged at 900 rpm for 5 min. Transfection was performed with the Rothifect/lipofectamine method according to the manufacture's manual (Carl Roth, Karlsruhe, Germany). After transfection cells were cultured under constant selection pressure with geneticin sulfate 418 (Sigma, St.Louis, USA).

### Expression vectors and knock down (KD) vector cloning

Reverse transcription-polymerase chain reaction was applied for full length cloning of xCT from rat mRNA samples. For sequence alignments and homology searches of xCT we utilized the www.ncbi.nlm.nih.gov database and A-Plasmid editor software (ApE; MW Davis, Utah, USA). The sequences of rat xCT are deposited at the NCBI database (GenBank accession no. NM001107673). For construct cloning we cloned fragments by PCR and inserted the resulting amplicons into the pEGFP (Takara, Heidelberg, Germany) vector. According to the critera of Ui-Tei et al. [22] three 19-mer short interfering RNAs were chosen for RNA interference with rat xCT transcripts. Cloning of the synthetic oligonucleotids into the pSuperGFP vector (pS-GFP; OligoEngine) was performed by digesting the empty vector with EcoR1 and Xho1 according to the manufacturer's instruction. Cells were transfected at low density (<20.000 cells/cm^2^) and expression analysis was performed according to the protocol described by Savaskan et al. [6].

### Vascular organotypic brain slice cultures (VOGIM) and angiogenesis analysis

Brain slice cultures were conducted with five days old Wistar rat pups. Brains were prepared and maintained as previously described [23]. Briefly, animals were sacrificed by quick head dissection and brains were removed and kept under ice-cold conditions. Frontal lobe and cerebellum were dissected of the hemispheres. The remaining brain was cut into 350 μm thick horizontal slices using a vibratome (Leica VT 1000S, Bensheim, Germany). The brain slices were thereafter transferred onto culture plate insert membrane dishes with a pore size of 0.4 μm (GreinerBioOne, Frickenhausen, Germany) and subsequently transferred into six-well culture dishes (GreinerBioOne) containing 1.2 ml culture medium (MEM–HBSS, 2∶1, 25% normal horse serum, 2% L-glutamine, 2.64 or 14.3 mg/ml glucose, 100 U/ml penicillin, 0.1 mg/ml streptomycin, 10 μg/ml insulin–transferrin–sodium selenite supplement and 0.8 μg/ml vitamin C). The slices were cultured in humidified atmosphere (35°C, 5% CO2). After 24 hs, the 6-well plates were washed with 1.2 ml PBS and a full culture medium exchange was performed and DEXA treatment was started. At the second day after preparation, 0.1 μl of the cell-medium-suspension was placed onto the cortex of the slice (10,000 F98 peGFP cells) by using a 1-μl-Hamilton-syringe.The medium was exchanged after implantation and from that time forward every other day over a course of 7 days, propidium iodide (PI) staining was performed at the same time of medium change, incubating slices with 1 μg/ml propidium iodide (PI) (Sigma) for 15–20 min. Afterwards slices were washed in 6-well plates with PBS. After washing with PBS, complete medium exchange followed. DEXA treatment was performed after complete medium change, 1.2 μl 1 mg/ml and 2 mg/ml DEXA were added to the new medium to obtain the final concentration of 100 μM and 200 μM: At the 7^th^ day of culture, all slices were fixed in immunofixative solution (containing of 4% formaldehyde and picric acid) and stained with antibodies directed against laminin (1∶250) (Sigma) or CD105 (1∶200) (Abcam) for vessel analysis. Vessel density quantification was performed by the overlay grid method [24] as described. In brief, we designed 80×80 μm grids to cover the entire vessel images and calculated the number of events in which single vessels crossed the grids.

### Cell death analysis

Cell death in living brain slices was evaluated by measuring PI signal intensity processed with the image J software (NIH, USA). For tumor-implanted slices, cell death was measured within the tumor region (identified by light microscopy or its GFP signal). Cell death in the peritumoral area was identified by the PI signal in the surrounding area of the tumor core. As control area we chose cortical regions distant to the tumor core and peritumoral region. For non-tumor implanted native slices, total cell death was evaluated by measuring the PI-signal of the whole cortical slice region.

### Primary rodent astrocyte and neuronal cultures

Primary rat astrocytes were isolated from rat whole brains aged P4–P6 without cerebellum. (Charles River Laboratories, Wilmington, MA). Brains relieved of meninges were placed in ice-cold HBSS buffer without serum. Then, tissue was further gently triturated with fire-polished Pasteur pipettes of diminishing tip diameter until tissue was uniform homogenized. Minced brain tissue was trypsinized with 0.25% trypsin for 10 min. After resuspending and centrifugation, astrocytes were resuspended in full DMEM medium (Biochrom, Berlin, Germany) supplemented with 10% fetal calf serum and penicillin/streptomycin (Biochrom). From the following day on astrocyte culture flasks were continuously agitated to separate detaching microglia. For determination purity, astrocytes were plated on non-covered glass slides and after 48 h culturing cells were fixed in 4% formalin for 15 min and processed for glial fibrillic acidic protein immunostaining (Dako, Hamburg, Germany). GFAP positive cells indicated a purity of rat astrocytes above 90%. Up to passage #7 astrocytes were used for DEXA experiments.

For the preparation of hippocampal neuronal cultures we used freshly isolated postnatal Wistar rat brains [25]. Hippocampi were removed from newborn rat brains in ice cold Hank's salt solution. After trypsin digestion neurons were triturated mechanically and plated in MEM medium, supplemented with 10% fetal calf serum and 2% B27 supplement (all from Life Technologies-Invitrogen, Karlsruhe, Germany). Treatment of neurons was started after 20 days in vitro (DIV).

### Primary human astrocytes

Primary human astrocytes were freshly isolated from optic nerve donors. For the establishment of astrocyte cultures, optic nerve head (ONH) tissue was dissected into six explants, placed into laminin-coated 6-well culture plates (BD Biosciences; Heidelberg, Germany), capped with a coverslip, and maintained in 2 ml astrocyte growth medium (AGM), DMEM/Ham's F12 with 10% fetal bovine serum, 1% antibiotic-antimycotic mix, Invitrogen; Karlsruhe, Germany), supplemented with 5 ng/ml bFGF and 5 ng/ml PDGF-AA (PAN-Biotech, Aidenbach, Germany) in a 95% air – 5% CO2 humidified atmosphere at 37°C. At the first passage, ONH astrocytes were separated from other cell types by selective adhesion to the growth surface as previously described [26]. In brief, ONH cells were trypsinized and plated in serum-free AGM for 24 h, other cell populations failed to attach in serum-free conditions and were removed with medium change. After resuspending and centrifugation, astrocytes were resuspended in full DMEM medium (Biochrom, Berlin, Germany) supplemented with 10% fetal calf serum and penicillin/streptomycin (Biochrom). Glial fibrillic acidic protein immunostaining (Dako, Hamburg, Germany) was used to facilitate the determination of astrocyte purity. Human astrocyte cultures revealed more than 90% GFAP immunopositive cells.

### Ethics Statement

Studies with human tissue wer conducted in compliance with the Helsinki Declaration and approved by the Ethics Committee of the Friedrich-Alexander-University of Erlangen-Nuremberg. All patients gave written informed consent to participate in the study.

Animal killing was performed in accordance with the German Protection of Animals Act §4 paragraph one and three. The announcement of rat and mouse killing was approved by the designee for animal protection of the University of Erlangen-Nuremberg (TS-7/12).

### Cell proliferation, apoptosis and cell cycle analysis

Cell proliferation was measured by using 3-(4,5-dimethylthiazol-2-yl)-2,5-diphenyl-tetrazolium-bromide (MTT) assay and propidium iodid (PI) staining. Cells were plated at 3000 cells/cm^2^ in 96-well plate and incubated at standard conditions. After 12 hs, the cells were treated with DEXA. At measure point (24 or 48 hs after treatment) cells were incubated with MTT solution (Roth, Karlsruhe, Germany) (5 mg/ml) or PI (Sigma) (3 μg/ml), at 37°C, 5%CO_2_. After 4 hs MTT incubation or 10 min PI incubation, pictures were taken with Olympus X71 microscope (Olympus, Hamburg, Germany) with 10× objective. Exposure time was equal in different groups. Images were taken with cell- F software (Olympus). Cells with 4 hs MTT incubation were then lysed with 100 μl Isopropanol+0.1 N HCl and OD value was measured with SLT spectra (Crailsheim, Germany) using Tecan×Fluor4 software. Apoptosis assays were performed with modifications as described in Halstead et al. (2006). Cells were plated at 100,000 cells/well in 6-well plates and incubated with DEXA for 2 days. Cells were trypsinized and fixed with 4% PFA on ice for 10 min, then spinned down at 900 rpm for 5 min. Cells were stained with HOECHST 33342 (Invitrogen, SIGMA) [c = 16 μg/ml] for 10 min in the dark. Suspension was added to an objective glass and mounted on cover slips Pictures were taken at ×200 with Olympus ×71. Nuclei fragmentation was counted and set related to total number of cells on image. For cell cycle analysis, we used the Nicoletti assay. In brief, cells were treated with DEXA in 6-well plates, after 48 hs treatment, cells were trypsinized and collected together with their supernatant, then spun down and resuspended in PBS. Cells were washed one more time and cells were collected by centrifugation. The supernatant was discarded and the pellets were resuspended in PI-LB (0.1% sodium citrate+0.1% Triton X-100+100 μg/ml RNAse+50 μg/ml propidium iodide). Cells were agitated gently and analysis was performed by flow cytometry (BD Biosciences, FACSCanto II Heidelberg, Germany) within the next 2 hs.

### RNA isolation and qRT-PCR experiments

Cells were scratched and collected in a 1.5 eppendorf tube after 6 hs of DEXA treatment in 6-well plates, followed by suspension in 200 μl PBS. Total RNA out of cells was extracted using High Pure RNA Isolation Kit (Roche, Mannheim, Germany) following the manual's description. RNA concentration was quantified by NanoVue Plus Spectrophotometer (GE Healthcare, UK). cDNA synthesis was performed with SuperScript III Reverse Transcriptase (Invitrogen). qRT-PCR was performed with SYBR Green PCR master mix (Qiagen). The oligos used in this study are: Rat xCT forward primer: TGCTGGCTTTTGTTCGAGTCT; Rat xCT reverse primer: GCAGTAGCTCCAGGGCGTA. Human xCT forward primer: CCCAGATATGCATCGTCCTT; Human xCT reverse primer: GCAACCATGAAGAGGCATGT. GAPDH forward primer: TGCACCACCAACTGCTTAGC; GAPDH reverse primer: GGCATGGACTGTGGTCATGA. Beta-actin forward primer: GCTCCTCCTGAGCGCAAG; Beta-actin reverse primer: CATCTGCTGGAAGGTGGACA. Real time cycling parameters: Initial activation step (95°C, 15 min), cycling step (denaturation 94°C, 15 s; annealing at 60°C, 30 s; and finally extension for 72°C, 30 s ×40 cycles), followed by a melting curve analysis to confirm specificity of the PCR. The Ct value was corrected by Ct reading of corresponding GAPDH or Beta-actin controls. Data from three determinations (means ± SEM) are expressed as relative expression level. The reaction was performed using Light Cycler 480 (Roche). The specificity of the PCR reaction was confirmed by agarose gel electrophoresis.

### Protein isolation and immunoblotting

Cells were maintained under standard conditions. For protein extraction samples were lysed with NP 40 buffer containing a protease inhibitor cocktail (Roche, Basel, Switzerland) and homogenized by ultrasound (Bandelin Sonoplus, at 67%). After 20 min on ice, samples were centrifuged at 8000 rpm for 8 min. Supernatants were measured with NanoVue Plus Spectrophotometer (GE Healthcare, UK). Samples were mixed with loading buffer (4×) and Reducing agent (10×) (Invitrogen, California, USA) and boiled at 96°C for 8 min. Equal amounts of protein sample were loaded on 4–12% SDS-NuPage Gel (Invitrogen, CA, USA) and electrophoresis was performed in MOPS-buffer, transferred on PVDF membranes (Roth, Karlsruhe, Germany) and efficiency was checked with Memcode Stain kit (Thermo Massachusetts, USA) according to the user manual. Membranes were blocked in PBS containing 2% Magic block and 10% Top block (Lubio science, Lucern, Switzerland) for 1 h before further processed. Antibodies were incubated overnight at 4°C in roller tubes, followed by secondary antibodies incubated at room temperature for 1 h. Detection was performed with ECL plus kit (GE-healthcare, Solingen, Germany).

### Amino acid profiling of glioma conditioned medium

Cells were seeded in 6-well plates at a density of 75.000 cells per well in full DMEM medium. For the experiments at least three wells per cell line were used and the assay was performed three times. After incubation overnight, the medium was changed to only DMEM without any supplements. After incubating for another 24 hs, medium was collected and measurement was performed by using high performance liquid chromatography (HPLC). Amino acids were analysed by ion-exchange chromatography and post-column ninhydrin derivatization technique using a fully automated amino acids analyzer (Biochrom 30+, Laborservice Onken, Gründau, Germany). For the amino acid analysis, 100 μL of sample was deproteinized with 100 μL of 10% sulphosalicylic acid. 20 μL of this supernatant was then loaded by the autosampler into a cation-exchange resin-filled column.

### Statistical significance

Data from experiments were obtained from at least three independent experiments (n≥3) if not otherwise stated. Statistical analysis was performed using MS Excel 2010 (Microsoft Corp., Washington, USA) if not otherwise specified in the figure legends. The level of significance was set at P*<0.05 according to the international conventions. Error bars represent ± S.D.

## Results

### Dexamethasone inhibits glioma growth in a species-specific manner

**Figure 1:**
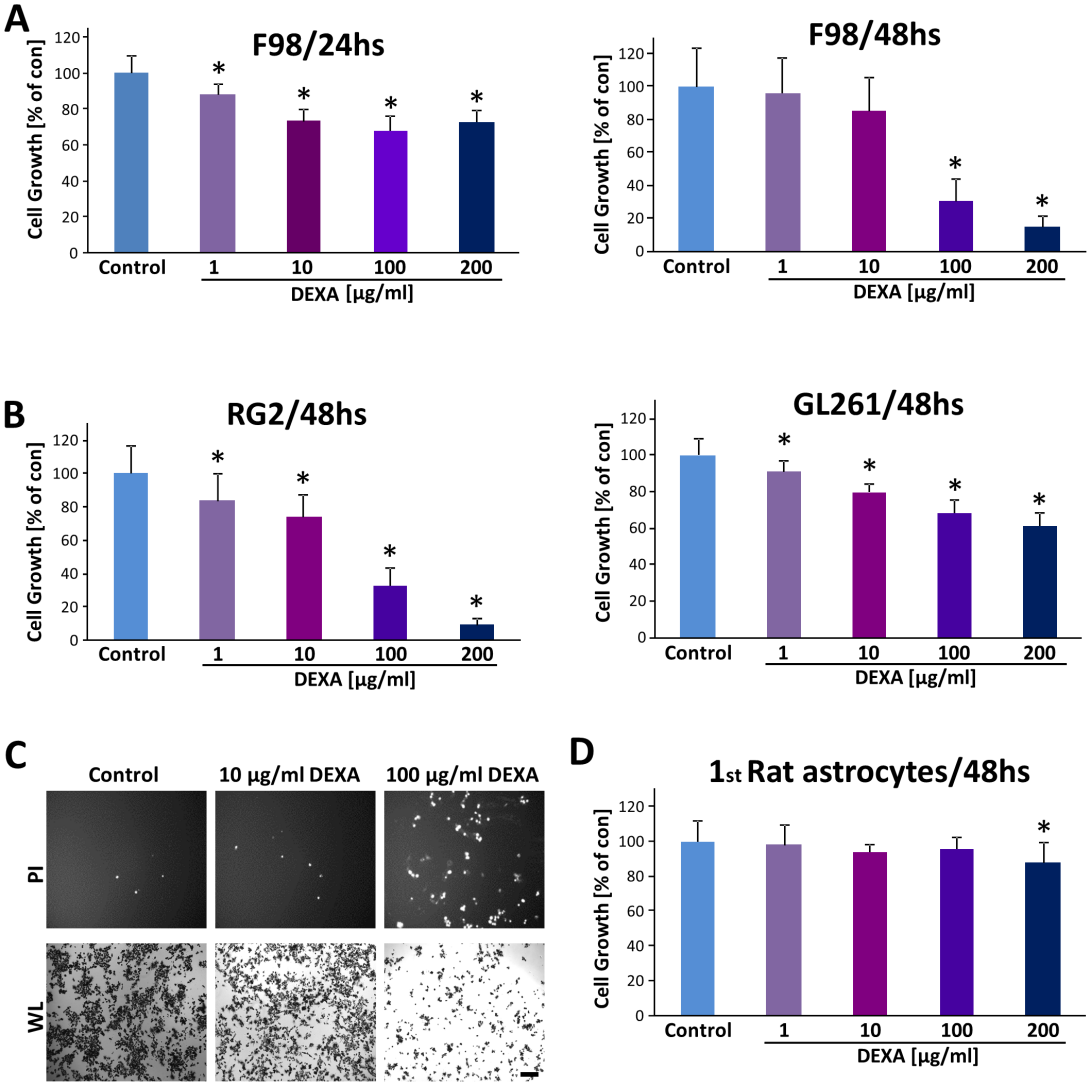
Dexamethasone promotes glioma cell death and growth inhibition. Different rodent and murine glioma cells (F98; RG2 and GL261) were treated with various concentrations of dexamethasone (DEXA) and proliferation was determined. (**A**), DEXA treatment of F98 glioma cells for 24 hs (left) and 48 hs (right). After the indicated time points, cell growth was determined. (**B**), DEXA treatment for duration of 48 hs in rodent RG2 glioma cells (left) and in murine GL261 glioma cells (right). Note, that low DEXA levels from 1 μg/ml onwards already reduce glioma proliferation in RG2 and GL261 cells. (**C**) Representative images of glioma cells treated with DEXA. Propidium iodide (PI) staining reveals dead cells (white dots, upper panel). Note the increase in PI-positive cells and reduced cell number under white light (WL) view in the groups treated with low or high dexamethasone. Scale bar represents 200 μm. (**D**) Dexamethasone does not alter the proliferation of rat primary astrocytes. DEXA was tested at concentrations ranging from 1 μg/ml to 200 μg/ml. Determination of cell death assays was repeated at least three times. Values are given as mean ± S.D. Quantification is given for n =  12. Statistical analysis was performed with Student's *t*-test (two-sided), asterisks indicate p-values <0.05 which were considered significantly different from control groups (set as 100%). Abbreviation: DEXA, Dexamethasone; PI, propidium iodide; WL, white light.

We first analysed the effect of dexamethasone on glioma cells. For this we measured proliferation of glioma cells under various concentrations of dexamethasone (DEXA) at different time points. Cell growth analysis revealed a 70% to 80% decrease of cell growth in rodent glioma cells (F98 and RG2 gliomas) after 48 hs' 100 μg/ml dexamethasone treatment (**Fig. 1A**). Noteworthy, RG2 and GL261 glioma cells appeared to be more sensitive to DEXA compared to F98 glioma cells (**Fig. 1B**). The growth inhibitory effect was observed already at the lowest concentration (1 μg/ml) (**Fig. 1A, B**). We could further confirm these results by monitoring cell death (propidium iodide (PI) staining) and white light morphological analysis (**Fig. 1C**). Dexamethasone treated gliomas had significantly more dead cells which were PI positive (red), and less living cells as revealed by morphological assessment.

We further tested whether DEXA is generally toxic to differentiated normal brain cells. For this we prepared primary rat astrocytes and applied DEXA at the same setting as in glioma cells. Noteworthy, DEXA showed no toxicity on primary astrocytes within a wide concentration range shown to be toxic for glioma cells (**Fig. 1C**).

**Figure 2:**
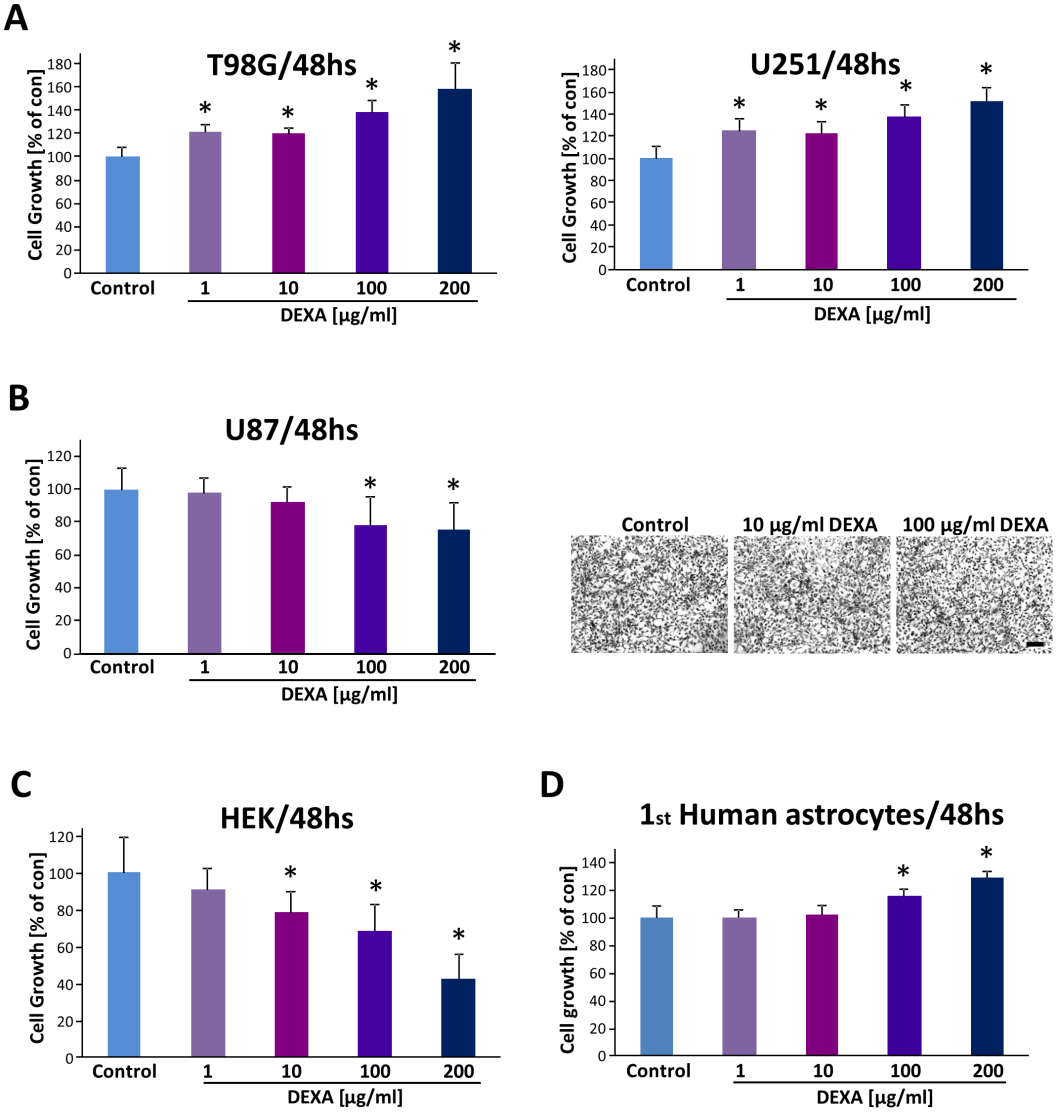
Dexamethasone acts differentially on human glioma cells. (**A**), Dexamethasone (DEXA) promotes human T98G and U251 glioma growth. Human T98G and U251 glioma cells were cultured at various concentrations of DEXA and cell viability was determined with the MTT assay. (**B**), Dexamethasone reduces human U87 glioma cell growth solely at high concentrations. Right, representative images of U87 glioma cells treated with low and high dosage of DEXA. Scale bar represents 200 μm. (**C**), Human embryonic kidney cells are growth sensitive to increasing DEXA concentration. (**D**), Human primary astrocytes are resistant to various DEXA concentrations. Cell growth is given in relation to untreated controls. Quantification is given for n = 12. Values are given as mean ± S.D. with controls set as 100%. Differences were considered statistically significant with p<0.05 (asterisks, two-sided Student's *t*-test).

We next tested the efficacy of DEXA on established human glioma cell lines. In T98G and U251 glioma cells DEXA did not affect cell proliferation and contrariwise human gliomas proliferated more under DEXA compared to untreated controls (**Fig. 2A**). Conversely, at higher concentrations DEXA even promoted glioma cell proliferation. Dose-response experiments revealed a 20 to 60% elevation of cell growth in human T98G and U251 glioma cells after DEXA treatment (**Fig. 2A**). Hence, we went on and investigated another well-established human glioma cell line (U87). DEXA application with up to 10 μg/ml did not inhibit U87 cell growth (**Fig. 2B**). At higher concentrations beginning with 100 μg/ml onwards DEXA was effective in reducing significantly U87 cell growth (**Fig. 2B**). To analyse the general toxic potency of DEXA, we treated human embryonic kidney cells (HEK cells) with various concentrations of DEXA. These experiments revealed that 1 μg/ml DEXA is already efficient to inhibit proliferation of HEK cells (**Fig. 2C**). Higher concentrations of DEXA were even more efficient in inducing HEK cell death (**Fig. 2C**). We further investigated the effects of DEXA on human non-transformed control cells. For this we utilized primary human astrocytes and treated them with DEXA at the same condition as in glioma cells. Human primary astrocytes showed to be resistant towards various DEXA levels (**Fig. 2D**). Altogether, these results demonstrate, that DEXA acts as a growth inhibitor on glioma cells in a species-specific manner with different dose-response efficacy.

### DEXA does not impact neuronal cell survival and induces growth arrest in gliomas

**Figure 3:**
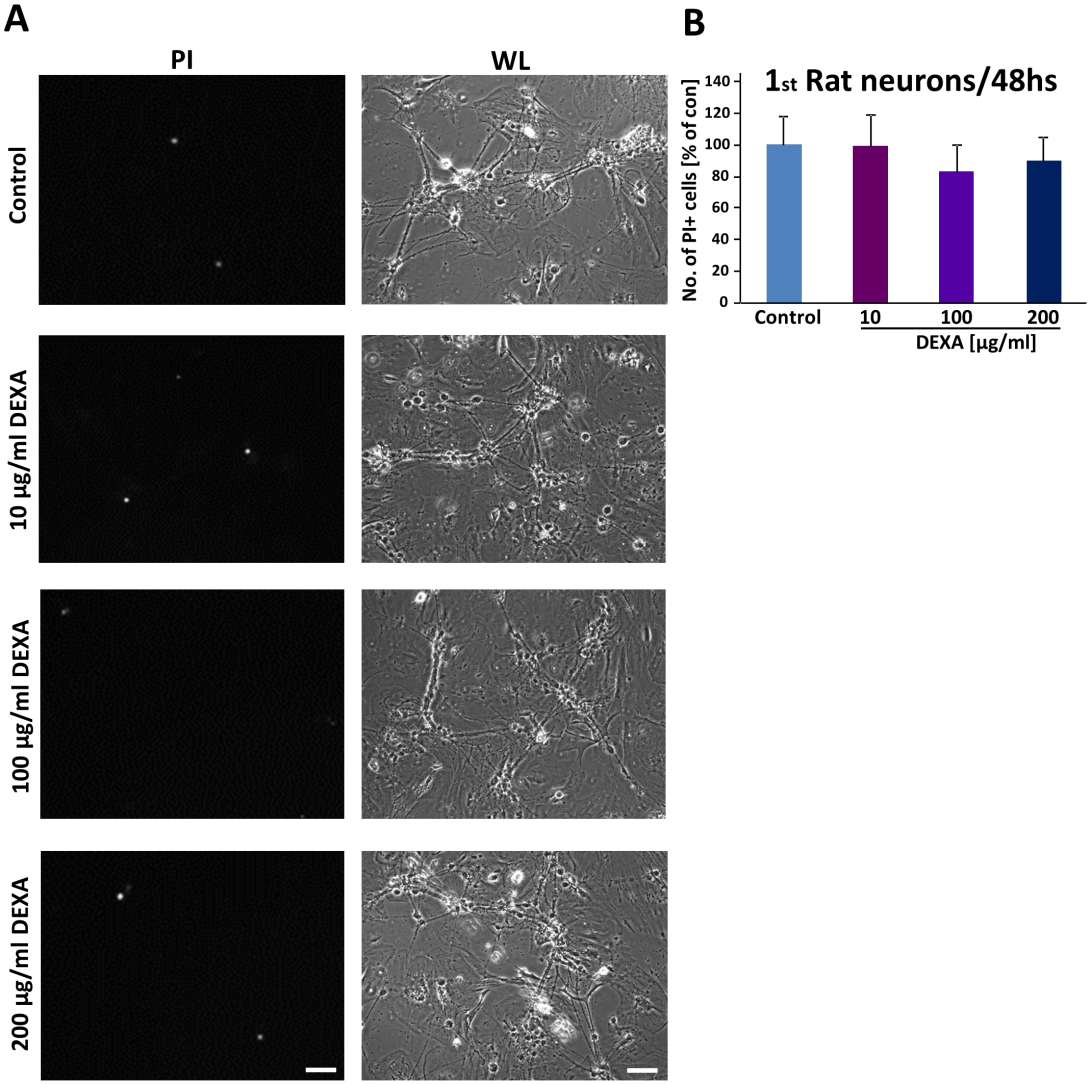
Dexamethasone does not affect cell viability of primary neurons. (**A**), Dexamethasone (DEXA) was applied to rodent hippocampal neurons for 48 hs and cell death was determined by PI staining (PI, propidium iodide, left column, Scale bar represents 200 μm). Right, white light microscopy indicates morphological integrity of neurons following DEXA treatment. Scale bar 100 μm (**B**), Neurons were cultured at various concentrations of DEXA for two days and cell viability was determined by measuring the PI signal intensity.

We next investigated the effect of dexamethasone on primary hippocampal neurons. For this we treated differentiated neurons with various concentrations of DEXA and monitored cell morphology and cell death (**Fig. 3A**). Interestingly, primary neurons appeared to be insensitive towards DEXA and cellular morphology was unaffected (**Fig. 3A**). We then assessed quantitatively cell death in primary neurons and found no significant challenges under DEXA treatment (**Fig. 3B**).

**Figure 4:**
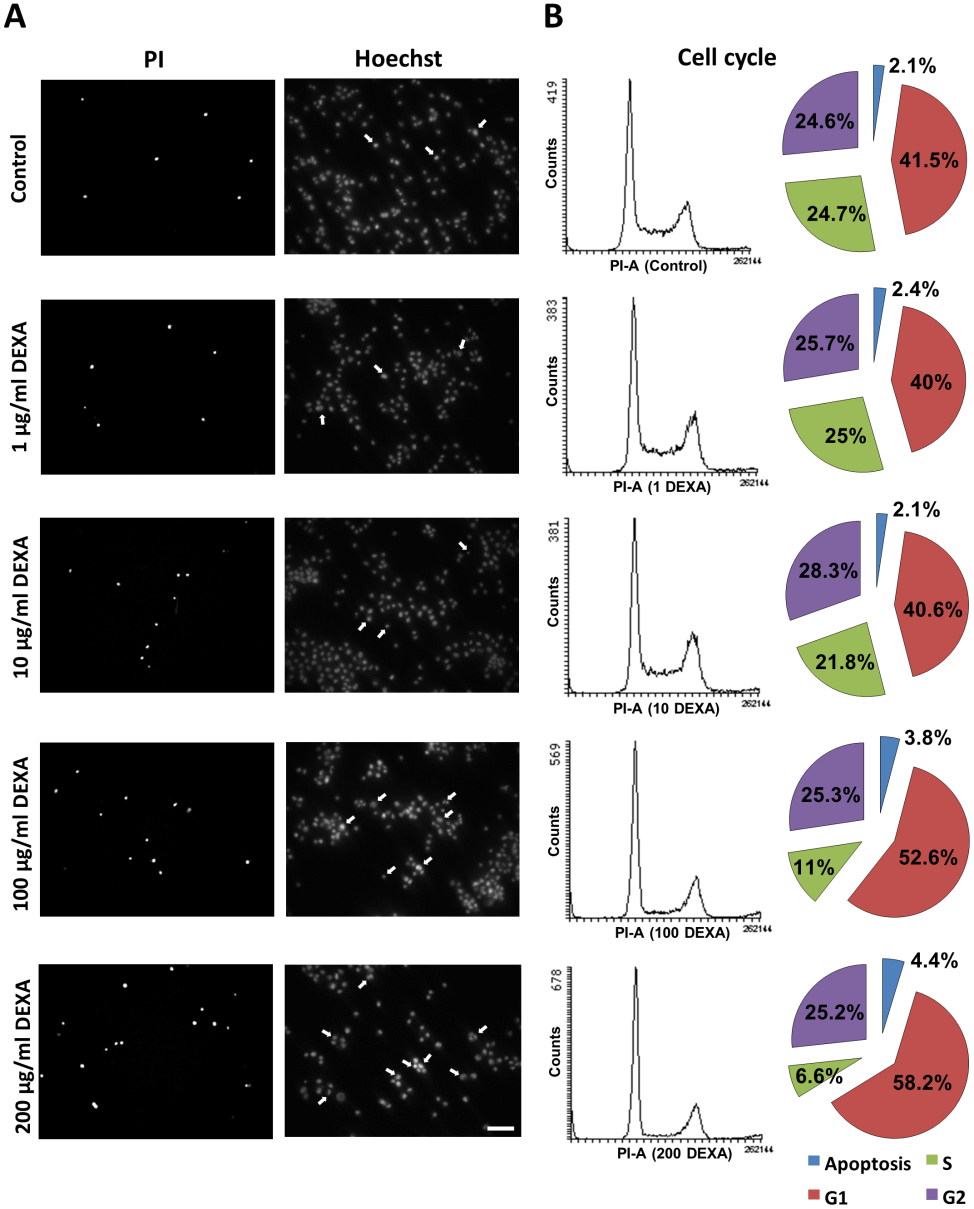
Dexamethasone alters the cell cycle profile of glioma cells. (**A**), Dexamethasone (DEXA) was applied to DEXA-sensitive F98 glioma cells for 48 hs and cell death was determined by PI staining and apoptosis was evaluated by HOECHST staining. Arrows indicate apoptotic nuclei defined as fragmented or irregular shaped. Scale bar represents 200 μm (**B**), Cell cycle analysis in rat glioma cells (F98) and following DEXA treatment at various concentrations. Representative profile graphs are shown on the left. Pie charts on the right show percentage of cells in different cell cycle phases (n = 3).

Hence, we analysed the mode of action of DEXA on glioma cells. For this we investigated glioma cells after DEXA treatment and then inspected these cells for morphological signs of apoptosis (**Fig. 4A**). In line with previous results DEXA treatment increased slightly the number apoptotic cells when applied with a concentration of 100 μg/ml or more (**Fig. 4A**). We next profiled the cell cycle of F98 glioma cells following DEXA treatment (**Fig. 4B**). Here we found that the proportion of cells in the S-phase diminished significantly under increasing levels of DEXA in favour of elevated G1 phase (**Fig. 4B**). Moreover, DEXA treatment significantly augmented the apoptotic sub-G1 fraction in glioma cells (**Fig. 4B**). Thus, DEXA induces glioma growth arrest and cell death in a concentration-dependent manner.

### DEXA promotes xCT and VEGFA expression in gliomas

**Figure 5:**
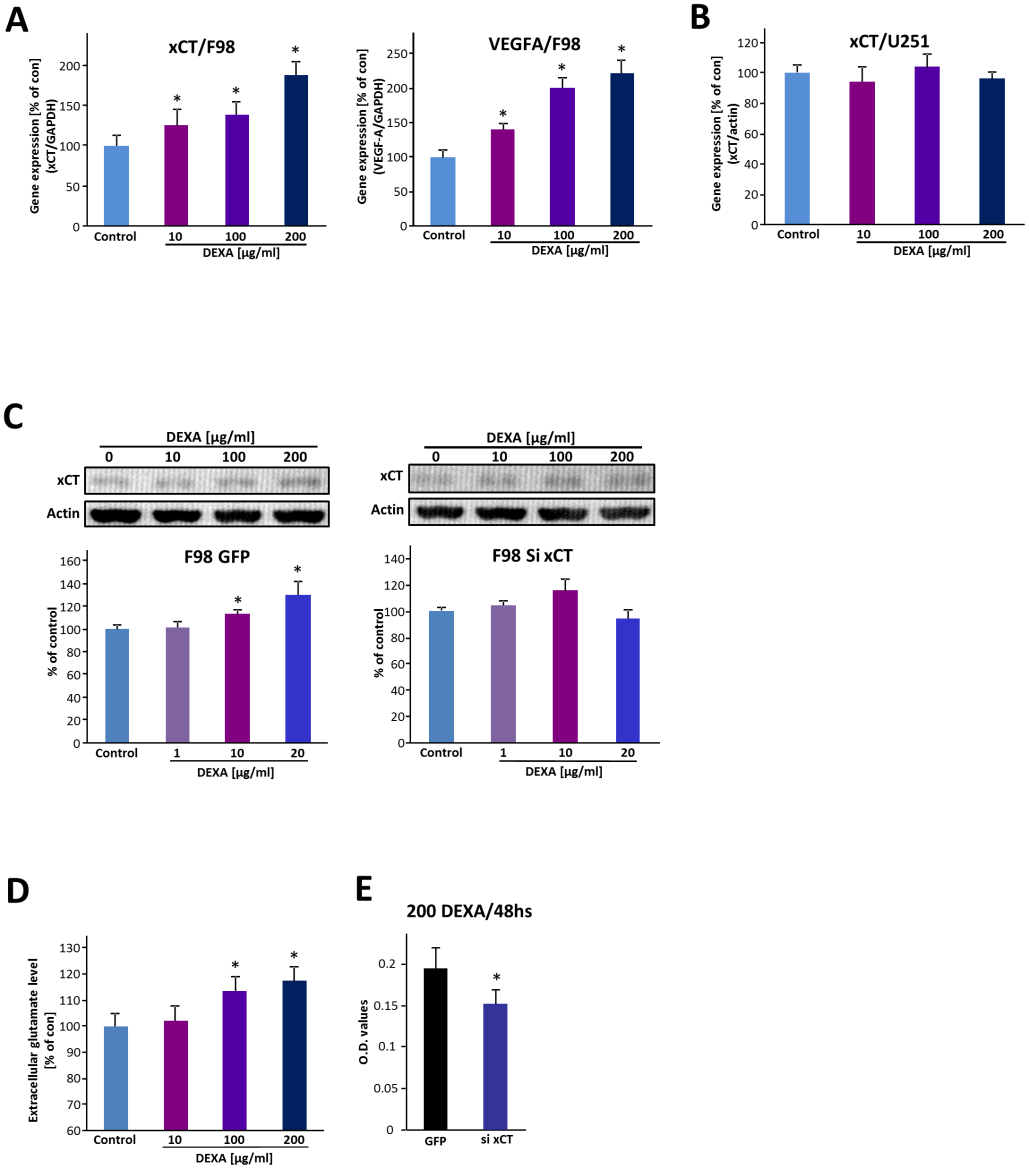
Dexamethasone differentially impacts stress responses in rat and human gliomas. (**A**), qRT-PCR analysis of F98 glioma cells treated with dexamethasone (DEXA) for 6 hs. DEXA induces upregulation of xCT and VEGFA expression in rat F98 gliomas. Quantification of RT-PCR results is given showing relative xCT mRNA (left) and VEGFA mRNA (right) values after DEXA treatment [0, 10, 100 and 200 μg/ml] in F98 glioma cells. (**B**) qRT-PCR analysis of DEXA-resistant U251 glioma cells. Note, that DEXA does not affect xCT mRNA expression in human U251 glioma cells. Quantification of the qRT-PCR results is depicted showing relative xCT mRNA level after DEXA treatment [0, 10, 100 and 200 μg/ml]. (**C**), Western blot analysis of xCT after DEXA treatment for 48 hs. Left, top, representative blot is given. Bottom, quantification of western blot (xCT/actin) showing that xCT is up-regulated in F98 glioma cells after DEXA treatment for 48 hs. Right, DEXA does not affect xCT expression in xCT silenced glioma cells. Top, Representative immunoblot of xCT in rat xCT knockdown gliomas after DEXA treatment. Bottom, quantification of western blot ratios (xCT/actin) after DEXA treatment for 48 hs. (**D**), Short-duration DEXA treatment enhances glutamate secretion in rat glioma cells. Glutamate concentration in condition medium 24 hs after DEXA application measured by ion-exchange chromatography with an amino acid analyzer reveals extracellular glutamate elevation after dexamethasone treatment. (**E**), siRNA mediated xCT knockdown increases glioma susceptibility to DEXA. DEXA treatment of control (GFP) transfected F98 glioma cells and xCT knockdown (si xCT) F98 cells. Two days after DEXA treatment, cell growth was determined. Means are given with quantification out of n≥3. Values are given as mean ± S.D. Control groups set as 100%. Differences were considered statistically significant with p<0.05 (asterisks, two-sided Student's *t*-test). Abbreviation: qRT-PCR, quantitative real-time reverse transcriptase PCR.

Since DEXA is a standard medical agent for the treatment of brain tumor-associated cerebral edema, we investigated the regulation of two genes associated with cytotoxic and vasogenic edema [7] [27]. We first tested the glutamate-cystine antiporter xCT/SLC7a11/system x_C_- in DEXA-responsive glioma cells. xCT is known to be associated with tumor-induced edema formation [6] as well as in hypoxia and redox-dependent stress responses [28] [29]. Interestingly, xCT mRNA expression is immediately elevated following 6 hs incubation with DEXA (**Fig. 5A**). Quantitative RT-PCR analysis further revealed that DEXA-induced xCT elevation is DEXA-regulated in a concentration-dependent manner (**Fig. 5A**). We next tested the expression of VEGFA under DEXA treatment since VEGFA is associated with tumor angiogenesis, cell stress, hypoxia and brain edema [30] [31]. Here, we found that glioma cells also up-regulate VEGFA following DEXA application in a concentration-dependent manner (**Fig. 5A**).

Since both edema-associated genes are also involved in hypoxia and stress-response conditions, we hypothesized that those glioma cells which are non-responsive towards DEXA treatment also do not challenge their xCT expression under DEXA conditions. For this we analysed xCT mRNA expression in U251 gliomas. In support of this hypothesis is the finding that the xCT mRNA expression remains unchanged following increasing concentrations of DEXA (**Fig. 5B**).

We next investigated the functional implication of xCT regulation in gliomas. First we tested the xCT protein levels after DEXA treatment. Following DEXA application at low concentrations gliomas enhanced the xCT protein expression (**Fig. 5C**). In xCT knock down gliomas, this DEXA-dependent up-regulation was absent (**Fig. 5C**). Since xCT operates as a glutamate exporter and glutathione regulator we analysed the functional consequences of DEXA treatment by determining the glutamate levels. Notwithstanding, short duration DEXA application for 24 hs elevated extracellular glutamate levels in a concentration-dependent manner (**Fig. 5D**). However, longer incubation times led to glioma cell death. Furthermore, we wanted to test whether the expression level of xCT influences the susceptibility for DEXA. For this we induced siRNA-mediated xCT knockdown in rodent glioma cells and analysed their proliferation. Interestingly, xCT knockdown increased glioma susceptibility towards DEXA treatment (**Fig. 5E**).

### Dexamethasone protects neurons and selectively kills gliomas *ex vivo*

**Figure 6:**
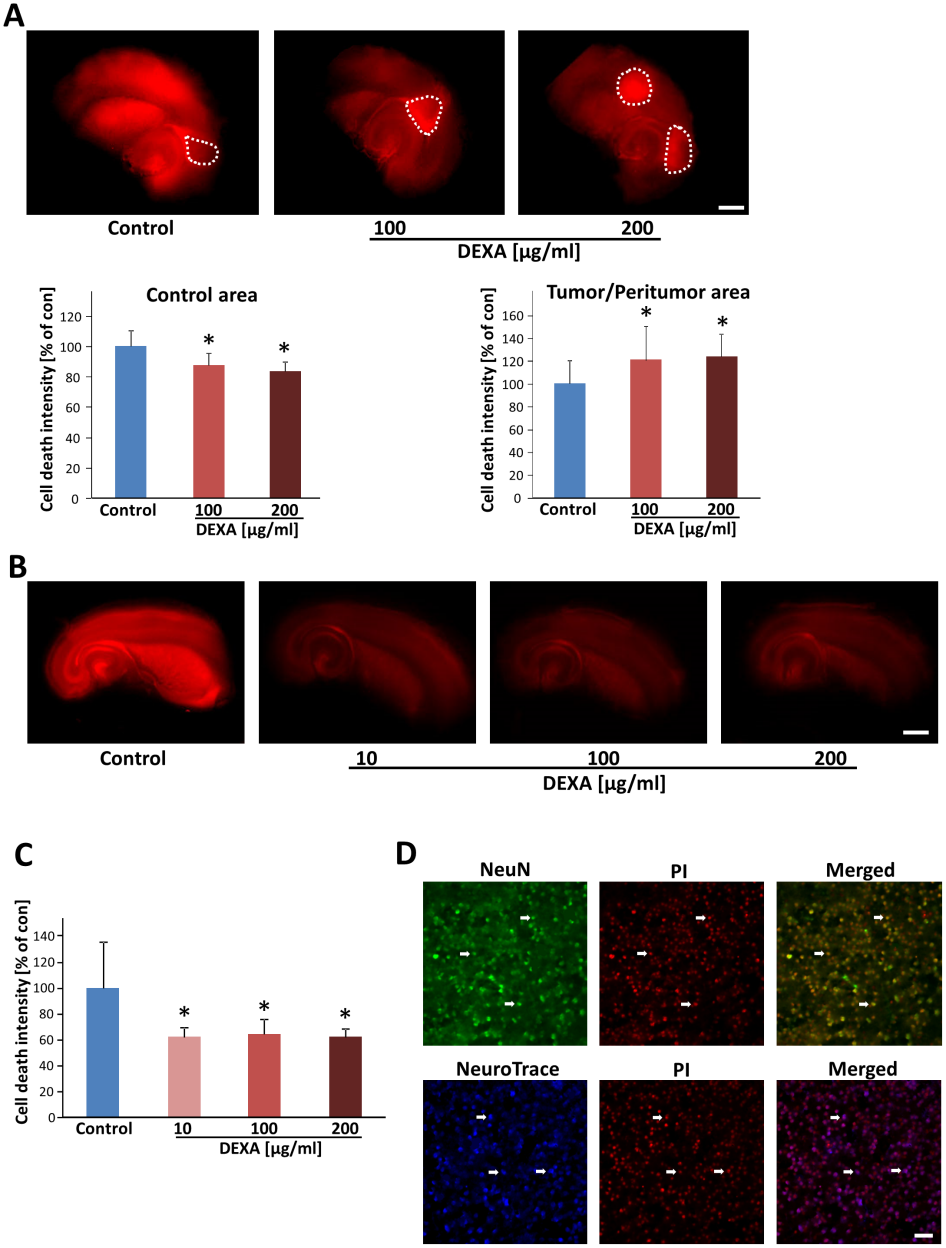
Dexamethasone induces tumor cell death in organotypic brain tissue. (**A**) Dexamethasone (DEXA) treatment induces tumor cell death in glioma cell implanted brain slices and reduces tumor-induced cell death. Top, F98 glioma cells were implanted in brain slices and after 8 days cell death was evaluated (propidium iodide positive cells are depicted in red). Bottom, Cell death quantification in untreated tumor-implanted brain tissue (control) reveals increased peritumoral cell death (peritumoral zone), whereas the tumor zone is spared of cell death. DEXA treatment reduces brain damage as revealed by reduced cell death in control cortical areas apart from the tumor, and in the peritumoral zones, while DEXA induces massive tumor cell death within the tumor core zone. Cell death intensity was quantified with NIH-*Image J* and for statistical analysis the t-test was applied. Means ± S.D. are given. *P<0.05, Student's *t*-test (two sided) (n = 12). The dashed circle marks the tumor core zone. Scale bar represents 1 mm. (**B**), DEXA treatment in native brain slices. Cell death quantification in untreated brain slices (control), and native brain slices with DEXA treatment at a concentration of 10 μg/ml, 100 μg/ml and 200 μg/ml. Scale bar represents 1 mm. (**C**), Assessment of cell death in native brain slices treated with DEXA. Cell death intensity was quantified with NIH-*Image J* and for statistical analysis the two tailed t-test was applied. Means ± S.D. are given. *P<0.05, Student's *t*-test (n≥3). (**D**); Glioma cells induced neuronal cell death. Tumor-implanted brain slices were stained for the neuronal markers NeuN (top, given in green) and NeuroTrace (bottom, depicted in blue) and cell death assessed by PI staining (red). Arrows showing that the dead cells (PI+) are mostly NeuN+ (green) or NeuroTrace+ (blue) neurons. Scale bar, 50 μm.

In order to test the effect of dexamethasone on brain tissues, we developed an *ex vivo* model which enables pharmacological testing in real time mode [32]. Brain sections were cut and slices were cultured on permeable PET membrane bathed in culture medium. Treatment was performed via adding DEXA into the medium. After five days in culture, cell death and cellular integrity were monitored in brain slices. DEXA-treated brain sections showed alleviated cell death compared with control brain tissue (**Fig. 6A**). We tested various concentrations of DEXA and at 100 μg/ml or more DEXA was effectively protective for brain tissue. Quantitative analysis showed a 36 to 38% decrease in neurodegeneration after DEXA treatment (**Fig. 6B**). After having established the neuroprotective effects of DEXA, we tested its potency in tumor-implanted brain slices. As expected from previous reports [33] [34] [35], implantation of glioma cells into brain slices led to markedly more neurodegeneration in peritumor area compared to untreated naïve slices (**Fig. 6C**). In contrast, in DEXA-treated groups, the tumor-induced neurodegeneration was significantly diminished (**Fig. 6C**). We further analysed the effects of DEXA on tumor growth. Here, gliomas showed enhanced tumor cell death after DEXA treatment, implicating that dexamethasone is not solely neuroprotective but at the same time acts also glioma-toxic in this *ex vivo* rodent tumor model (**Fig. 6C**). Quantification of cell death revealed an up to 16% reduction in cell death in control area and an increase of 22% of tumor cell death after dexamethasone treatment (**Fig. 6C**). Hence, we asked whether glioma-induced cell death operates mainly on neuronal cells (**Fig. 6D**). For this we stained tumor-implanted brain slices for neuronal markers such as NeuN and NeuroTrace and monitored the correlation to PI positive dead cells. As previously reported, most of tumor-induced cell death occurred in neurons (**Fig. 6D**) [34].

### Dexamethasone reduces tumor vascular density *ex vivo*

**Figure 7:**
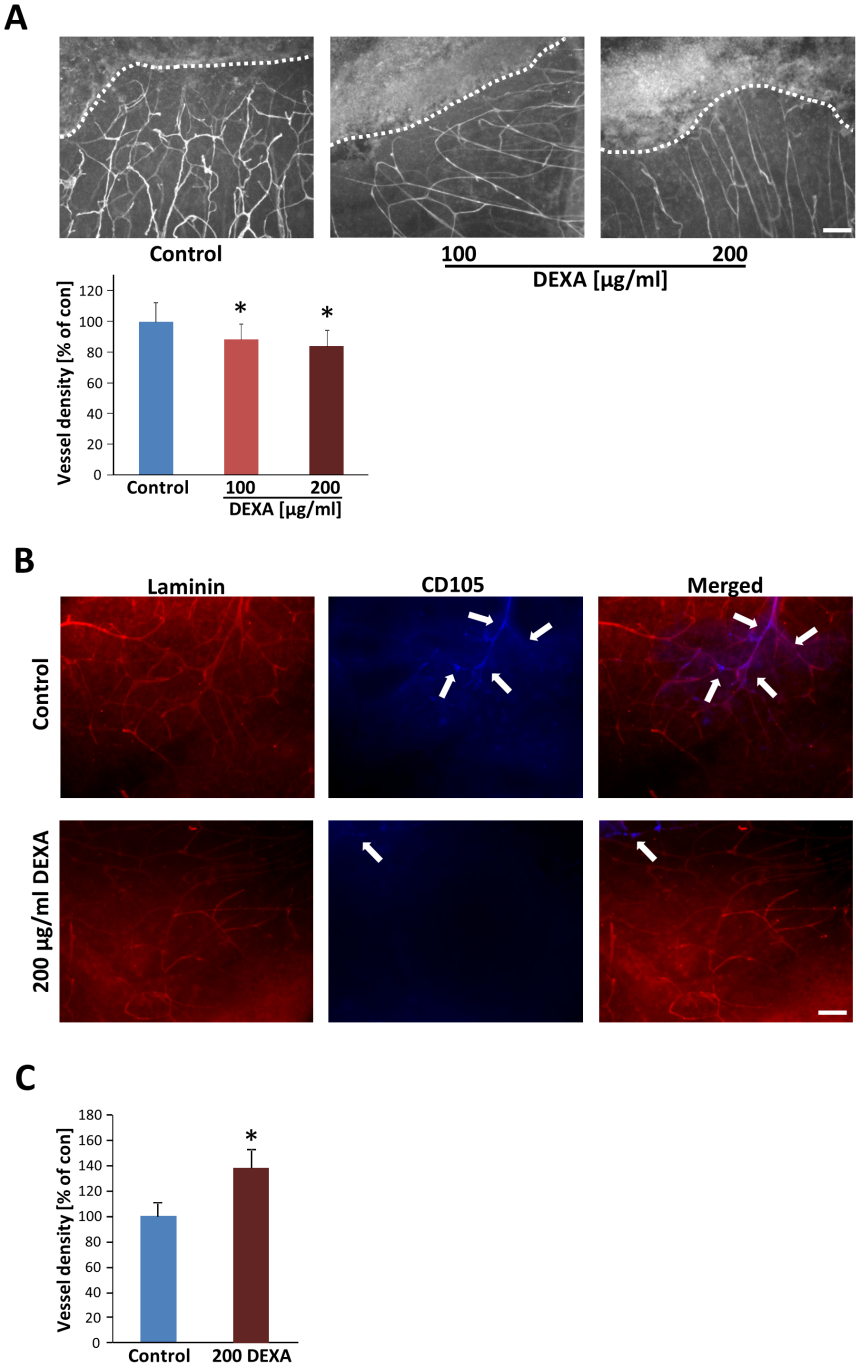
Dexamethasone inhibits specifically tumor angiogenesis. (**A**) DEXA treatment reduces tumor-induced vascular density. Top, tumor-implanted brain slices were analysed for vessels and morphological differences between tumor-implanted control groups (control) and dexamethasone treated groups were investigated. The dashed line designates the tumor core (top) from the peritumoral area (bottom). Vessel density in peritumoral area is reduced after 100 and 200 μg/ml DEXA treatment. Scale bar represents 100 μm. Bottom, quantitative analysis of vessel density in untreated controls and DEXA treated brain slices. Means are given with ± S.D. For statistical analysis the two tailed Student's *t*-test was applied and revealed *P<0.05 and n≥3. (**B**) DEXA treatment reduces newly formed tumor-induced vessels. Peritumoral area of tumor-implanted brain slices were analysed for vessels and differences in CD105 expression between tumor-implanted control groups (control) and dexamethasone treated groups were investigated. Laminin positive vessel structure is given in red (Left). CD105 positive endothelial cells are depicted in blue (middle). Merged images are shown on the right. Arrows indicate CD105 positive proliferating or newly formed endothelial cells. Scale bar represents 100 μm. (**C**) DEXA does not inhibit normal vessel growth. Native brain slices were treated with DEXA and vascular density was investigated. Quantitative analysis of vessel density in untreated brain slices (control) and DEXA treated brain slices. Scale bar, 50 μm. Means ± S.D. are given. For statistical analysis the two tailed Student's *t*-test was applied with *P<0.05 and n≥3.

It has previously been demonstrated that in gliomas cells are nourished by pre-existing blood vessels, which undergo hyper-angiogenesis [36] [37] [38]. Furthermore, these tumor-induced vessels are constantly changing in morphology and in function [39]. Therefore, we investigated tumor-induced vessels in our DEXA paradigm. As shown in **Fig. 7A**, wild type gliomas show irregular and hyper-vascularised spots and capillaries, often irregularly shaped with diameter leaps in contrast to vessels in non-infiltrated brain slice areas. Since these vascular challenges are presumably important for glioma growth, we asked whether dexamethasone could normalize certain vascular parameters, such as capillary density and morphology. To answer this question, we analysed the vessel architecture of tumor implanted brain slices after 10 days in culture. Through these investigations we found that DEXA-treated brain slices presented fewer capillaries in tumor and peritumoral areas (**Fig. 7A**). Beside this, DEXA-treated tumors showed fewer branches and fewer abnormal tortuous vessels resembling a more physiological phenotype (**Fig. 7A**). Through the overlay grid method [24], we found that the vascular density in peritumoral areas is moderately decreased in dexamethasone-treated brain slices compared to non-treated slices. Interestingly, newly formed vessels can be detected with the marker CD105. Therefore, we assessed whether DEXA operates specifically on tumor-induced vessels. Noteworthy, the proportion of CD105+ cells in the tumor microenvironment was alleviated when DEXA was applied (**Fig. 7B**). We further investigated the general effects of DEXA on the brain vasculature. For this we treated naïve brain slices with high concentrations of DEXA and monitored the vessel density. Noteworthy, DEXA did not reduce vascular density in normal brain tissue indicating that DEXA-induced tumor vessel regression is not a generally endothelial-toxic effect (**Fig. 7C**). Altogether, these results suggest that dexamethasone can also act on pathological brain capillaries and normalizes vascular density in brain tumors.

## Discussion

Dexamethasone is one of the most potent glucocorticoids available in neurooncology. This class of drugs has been applied for more than four decades in the treatment of brain tumor patients and belongs to the most powerful agents in minimizing tumor-associated brain edema. DEXA is still the drug of choice in neurooncology due to the lack of clinically approved alternatives. Conversely, there are various clinical schemes of optimal dosage of dexamethasone in glioblastoma patients [40] [41]. Unequivocally, the efficiency of DEXA and glucocorticoids in reducing the tumor-associated edema is nowadays overall clinically established [42] [12] [15].

Our initial hypothesis was that dexamethasone may affect glioma growth species-specific and regulate key factors of edema formation, namely xCT and VEGFA expression [6] [21]. Interestingly, our work unravelled that DEXA is efficient and selective in inducing glioma cell death whereas astrocytes are insensitive towards glucocorticoid treatment. Moreover, we could demonstrate that not all well-established glioma cells are affected by DEXA in the same way. There are mainly some human glioma cells such as T98G cells and U251 gliomas which are highly resistant towards DEXA treatment, although human U87 gliomas show a selective toxicity profile under DEXA, although weaker than in murine or rodent gliomas. Thus, DEXA is not generally ineffective on human cells. These findings may further explain some controversy in the field concerning anti-proliferative effects and ineffectiveness of DEXA in some patients [17] [18] [43]. Recent studies have indicated that malignant gliomas are heterogeneous allowing sub-classifying histologically identical GBM in to three or more subgroups due to their methylation status and expression profile. This genetic heterogeneity may be reflected by functional responses to DEXA [44] [45]. Generally, DEXA is toxic in human malignant cells such as in lymphocytic leukemia [46] [47]. The mechanism by which dexamethasone interferes with cell proliferation is not entirely uncovered. Glucocorticoids can challenge apoptotic and metabolic gene expression pattern with effects on key regulators such as IκB, Autotaxin (NPP-2), pyruvate kinase M2 (PKM2), and protein phosphatases [43] [48] [47]. Thus, glucocorticoids affect energy deprivation responses in malignant and highly proliferative cells [47] but not in proliferating primary astrocytes. This is also reflected by our xCT and VEGFA expression data. Although DEXA is operating anti-angiogenetic, both xCT and VEGFA expression are elevated under DEXA treatment in glucocorticoid-sensitive gliomas. These effects indicate redox-associated stress responses rather than constant pro-angiogenic signalling events [28]. In support of this are former reports, demonstrating DEXA as a VEGFA suppressor in gliomas [49]. Noteworthy is the finding that the gene xCT is not challenged through DEXA in resistant gliomas. The mechanism of DEXA-dependent gene regulation and cellular DEXA resistance is not known yet. Generally, glucocorticoids can transcriptionally regulate target genes via glucocorticoid receptor-dependent mechanisms, directly at the side of promoters as well as indirectly through the regulation of microRNAs [50]. Whether gliomas differ in their glucocorticoid receptor expression, promoter structures and microRNA make up need to be shown in future studies.

Another key finding is that DEXA exerts neuroprotective effects in the context of brain tumors. This is in particular of importance since tumor-induced neuronal cell death contributes to malignant cytotoxic brain edema. Relevant to this are independent findings of DEXA as a neuroprotective agent in newborn and adult hypoxia [51] [52] [53] [54] [55] [56]. Interestingly, DEXA's VEGFA enhancing effects on postnatal brain tissue has been attributed to this neuroprotection which is likely glucocorticoid receptor dependent [57]. Thus, at least in short duration DEXA is an efficient neuroprotective agent as well. Unfortunately, long-term usage of corticosteroids is associated with numerous well-characterized adverse effects such as diabetes, hyperlipidemia and psychosis. This promoted the search for alternative edema combating pharmaceutics. New antiangiogenic agents, such as bevacizumab (Avastin) are associated with significant steroid-sparing effects, allowing neuro-oncologists to reduce the overall use of corticosteroids in patients with progressive malignant brain tumors [58] [59]. However, bevacizumab is associated with potentiation of tumor-cell invasion and rapid progression following the cessation of therapy [60] [61]. This finding is of relevance since anti-angiogenic factors may contribute in addition to the edema-reducing condition. Vice versa, we and others proved that DEXA is efficient in reducing tumor-angiogenesis [43] [62]. However, concerning xCT inhibition clinical data are still far from complete to allow a definitive evaluation.

In conclusion, we could show that DEXA affects glioma cell growth differentially in a concentration-dependent manner. Further, DEXA exerts neuroprotective effects in the brain and reduces tumor-induced angiogenesis. Thus, our study shows that DEXA's pleiotropic actions affect differentially human tumor growth, tumor angiogenesis and neuronal survival.
